# Assessment of CHA_2_DS_2_-VASc score for predicting cardiovascular and cerebrovascular outcomes in acute myocardial infarction patients

**DOI:** 10.1097/MD.0000000000011230

**Published:** 2018-07-13

**Authors:** Chen-Yu Li, Chee-Jen Chang, Wen-Jung Chung, Cheng-Jui Lin, Shu-Kai Hsueh, Chien-Ho Lee, Chiung-Jen Wu, Tzu-Hsien Tsai, Cheng-I Cheng

**Affiliations:** a Clinical Informatics and Medical Statistics Research Center; b Foreign Language and International Trade School, Wenzhou Business College, Wenzhou, China; c Graduate Institute of Clinical Medical Science; d Research Services Center For Health Information Resource Center for Clinical Research, Chang Gung University, Taoyuan; e Chang Gung University College of Medicine; f Division of Cardiology, Department of Internal Medicine, Kaohsiung Chang Gung Memorial Hospital, Kaohsiung, Taiwan, R.O.C.

**Keywords:** acute myocardial infarction, atrial fibrillation, CHA_2_DS_2_-VASc score

## Abstract

Although established guidelines currently recommend the use of the CHA_2_DS_2_-VASc score for evaluating embolic risk in AF patients, few studies have evaluated the use of the CHA_2_DS_2_-VASc score for predicting cardiovascular outcomes in patients with acute myocardial infarction (AMI). The aim of this study was to determine whether CHA_2_DS_2_-VASc score is a predictor of a major adverse cardiocerebral vascular event (MACCE) in AMI patients.

This study analyzed data in the Taiwan National Health Insurance Research Database from January 2008 to December 2012. Cardiovascular outcomes were analyzed according to the baseline characteristics, presence of AF, and CHA_2_DS_2_-VASc score.

Twenty nine thousand four hundred fifty-two patients with non-fatal AMI, 1171 patients (8.3%) were with AF. The Cox regress model showed with the exception of women sex and peripheral artery disease, all the baseline characteristics considered risks in CHA_2_DS_2_-VASc scores were independently associated with the increased incidence of MACCE within 1 year after AMI. A CHA_2_DS_2_-VASc score of <5 had negative predictive values of 93.37% for recurrent MI, 98.45% for stroke, 94.86% for HF admission, 98.83% for mortality, and 87.80% for MACCE. Regardless of the presence of AF, the CHA_2_DS_2_-VASc score was correlated with 1-year MACCE.

The CHA_2_DS_2_-VASc score was correlated with 1-year MACCE in AMI patients who were discharge alive. The CHA_2_DS_2_-VASc score is useful predictor for 1 year MACCE in patients with AMI.

## 1 Introduction

Acute myocardial infarction (AMI) is the leading cause of death worldwide. Risk stratification using readily available clinical variables may be helpful for identifying risk subgroups for a major adverse cardiocerebral vascular event (MACCE) after AMI and for designing clinical managements for patients with specific clinical risks. Indeed, several clinical risk scores, including global registry of acute coronary events (GRACE) risk score^[1]^ and thrombolysis in myocardial infarction (TIMI) risk score^[2]^ are now widely used to assess future cardiovascular risk in AMI patients during hospitalization and during long-term follow-up. However, both scoring systems include laboratory parameters and focus on in-hospital outcomes.

An atrial fibrillation (AF)-associated coronary embolism may lead to type 2 AMI^[3]^ and is considered a risk factor for AMI.^[4,5]^ On the other hand, AF is a common and severe complication of AMI.^[6]^ Although new onset-AF may affect long-term outcomes of AMI,^[7]^ clinical predictors for new-onset AF after AMI are rarely investigated. Currently, the recommended guideline for evaluating embolic risk in AF patients is the CHA_2_DS_2_-VASc score, which consists of congestive heart failure, hypertension, age, diabetes, stroke/transient ischemic attack/thromboembolism, vascular disease, and sex.^[8,9]^ A recent study showed that the CHA_2_DS_2_-VASc score accurately predicts adverse events after acute coronary syndrome (ACS).^[10]^ However, large-scale studies of the use of the CHA_2_DS_2_-VASc score for predicting cardiovascular outcomes in AMI patients are rarely performed. Therefore, the aim of this study was to evaluate the use of CHA_2_DS_2_-VASc score for predicting MACCE in AMI survivors with or without AF.

## 2 Methods

The National Health Insurance system implemented in Taiwan in March, 1995 currently provides low-cost insurance coverage and high quality healthcare to approximately 99% of the Taiwan population. The National Health Insurance Research Database (NHIRD) established in 1996 contains data collected from 97% of Taiwan hospitals and clinics.^[11]^ Hence, the massive data contained in the NHIRD provide a complete history of diseases in Taiwan.

The protocol for this analysis of NHIRD data was approved by the Institutional Review Board of Chang Gung Memorial Hospital (102-3429B). Informed consent was not required as all patient information obtained from this secondary database was anonymized and de-identified before analysis.

Since the data available in the NHIRD were limited, the etiology of out-of-hospital cardiac arrest may not be identified correctly. Additionally, the ICD-9 codes may have been incorrect for AMI patient who had been discharged from the emergency department (ED) without hospitalization. Therefore, to avoid coding errors and to exclude the non-coronary etiology of out-of-hospital cardiac arrest, the analysis was limited to non-fatal AMI patients who had been hospitalized via the ED and discharged alive. The inclusion criteria in this analysis were admission for AMI and treatment by the ED of a regional hospital or medical center, age 18 years or older, discharge alive with a primary diagnosis of AMI with code 410 of the International Classification of Diseases, Ninth Revision (ICD-9), Clinical Modification (CM), and an NHIRD entry dated between January 1, 2008 and December 31, 2011.

To simplify analysis of the relationship between AMI and AF, the analysis excluded patients who had thyroid disease (ICD-9 codes 193, 240.9, 242.9, 244.9, and 648.13). The selected patients were then divided into 2 groups for further analysis: AMI with or without AF (ICD-9 code 427.31). Patient data collected from the NHIRD also included age, medical costs, and other treatment information.

The primary endpoint was defined as the occurrence of MACCE, including recurrent MI, admission for heart failure (HF) (ICD-9-CM code 428.0-428.1), cerebrovascular accident (ICD-9-CM codes 430–437), or death. Mortality related to MACCE was identified using death certificate data files with any diagnosis code, which also indicated the causes of death related to cardiovascular events.

Data were calculated as means or percentages. Chi-square test and Student *t* test were used to compare outcomes between AF and non-AF groups in the AMI survivors. The Cox proportional hazard model was used to estimate the hazard ratio of risk factors associated with MACCE. Tendencies in the occurrence of various cardiovascular events in the patient cohort were identified by linear-log regression analyses of CHA_2_DS_2_-VASc scores. The Kaplan–Meier method was used to estimate overall survival, and the log-rank test was used to compare treatments. All analyses were performed with SAS statistical software, version 9.4 (SAS Institute Inc., Cary, NC). A *P* value <.05 was considered statistically significant.

## 3 Results

**Figure 1:**
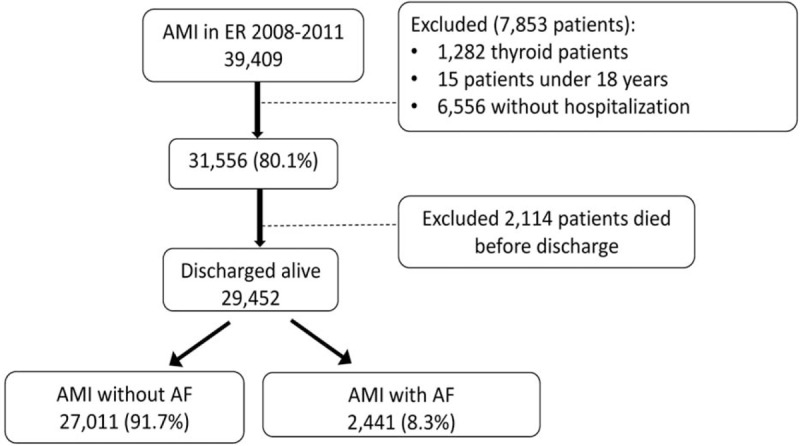
Selection of patients from NHIRD. The study population of patients treated for acute myocardial infraction with and without atrial fibrillation was selected from the 2008 to 2001 NHIRD. NHIRD = National Health Insurance Research Database.

After excluding 2114 (6.7%) patients who died in the ED or during hospitalization, the study population comprised 29,452 non-fatal AMI patients aged 18 years or older. The NHIRD data collected for these patients during 1 year after discharge were analyzed. Figure 1 shows that, of the 29,452 patients with non-fatal AMI, 2441 (8.3%) patients had a diagnosis of AF at discharge, which was considered coexisting with AF after the index event.

Table 1 shows the baseline characteristics of non-fatal MI patients with or without AF. Compared with the non-AF group, the AF group had a significantly older age and had significantly higher incidences of hypertension, chronic kidney disease, 3 chronic obstructive pulmonary disease, prior MI, prior stroke, HF, and peripheral arterial obstructive disease. The percentage of men and the incidences of dyslipidemia were significantly lower in the AF group. Table 2 shows that the AF group also had a higher incidence of treatment with warfarin, non-dihydrodipine calcium channel blockers, diuretics, or anti-arrhythmic drugs such as amiodarone or propafenone. In contrast, the AF group had a lower incidence of treatment with aspirin clopidogrel, beta blocker, and lipid lowering agents. Prescriptions for treatment with aspirin, angiotensin-converting enzyme inhibitor/angiotensin receptor blocker did not significantly differ.

**Table 1:**
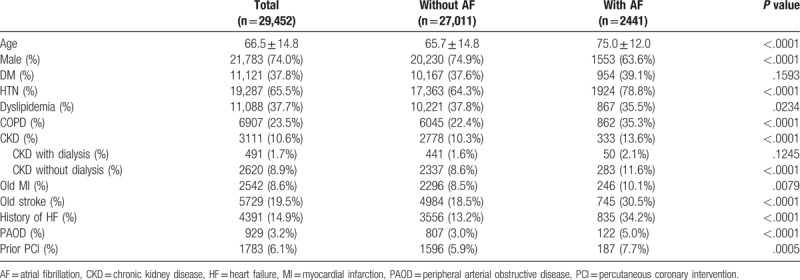
Baseline characteristics of study population stratified according to diagnosis of AF at discharge.

**Table 2:**
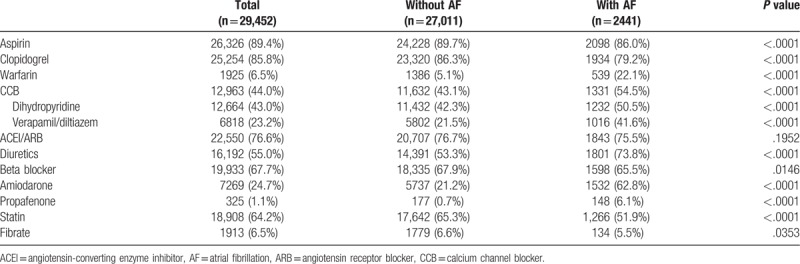
Cardiovascular medications during hospitalization.

**Figure 2:**
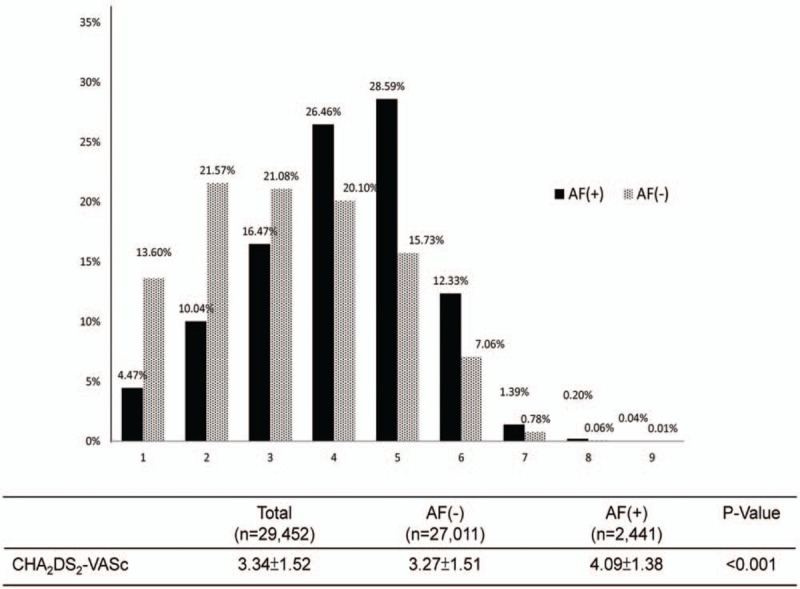
Distribution of CHA_2_DS_2_-VASc scores. The bar chart compares the distribution of CHA2DS2-VASc scores between non-AF (gray) and new-onset AF (black) patients. The numbers above the bars denote the respective percentages. The figure shows the average CHA2DS2-VASc score and *P* value for each group. AF = atrial fibrillation.

Mean CHA_2_DS_2_-VASc scores significantly differed between the AF group and the non-AF group (4.09 ± 1.38 and 3.27 ± 1.51, respectively; *P* < .001). Figure 2 shows the distribution of CHA_2_DS_2_-VASc scores for each group. That is, CHA_2_DS_2_-VASc scores tended to be higher in AMI patients with AF compared with AMI patients without AF.

To investigate the impact of the components of CHA_2_DS_2_-VASc scores on the long-term prognosis of AMI survivors, a multivariate Cox proportional hazards model was used to explore the incidence of MACCE (recurrent MI, HF admission, stroke, and all-cause death) during the year after discharge (Table 3). With the exception of woman sex and peripheral artery disease, all the baseline characteristics considered risks in CHA_2_DS_2_-VASc scores were independently associated with the increased incidence of MACCE within 1 year after AMI. Additionally, new-onset AF is also a significant predictor of MACCE [HR (hazard ration): 1.287; 95% confident interval [CI]: 1148–1.442; *P* < .0001]. Diagnosis of chronic kidney disease but not dyslipidemia was associated with risk of MACCE. Interestingly, women patients had significantly low risk of MACCE at 1 year (HR: 0.913; 95% CI: 0.861–0.968; *P* = .0022).

**Table 3:**
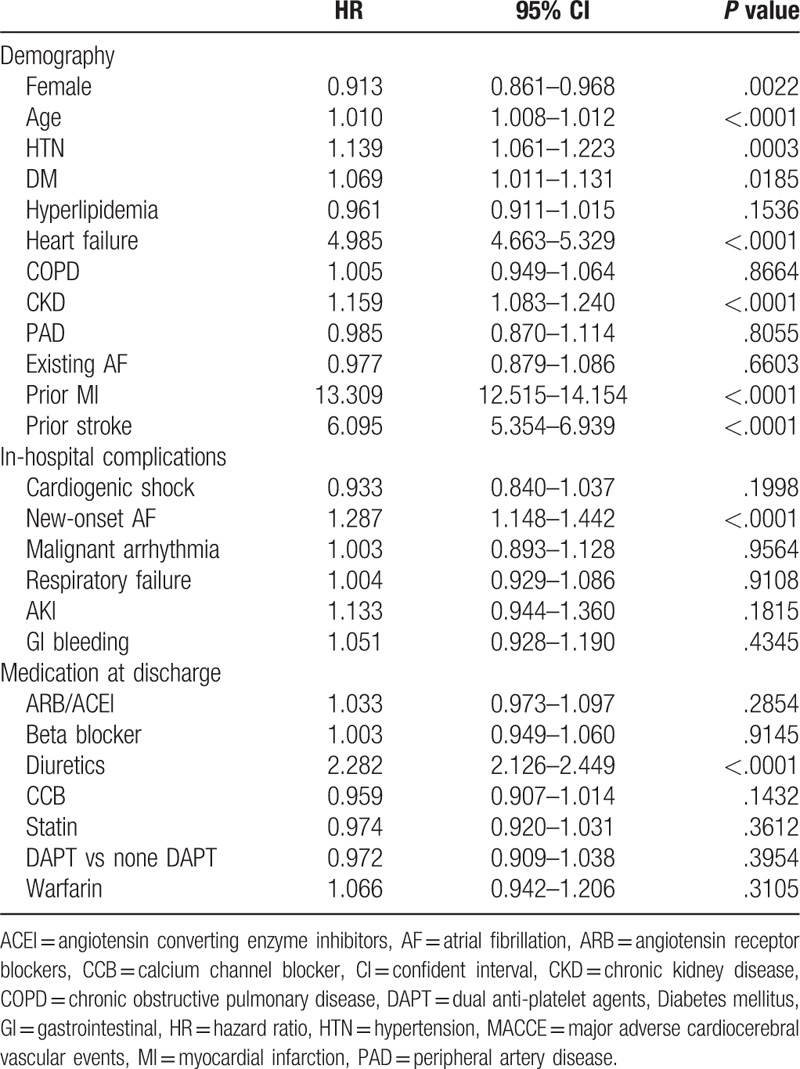
Multivariate Cox model for parameters associated with MACCE.

**Figure 3:**
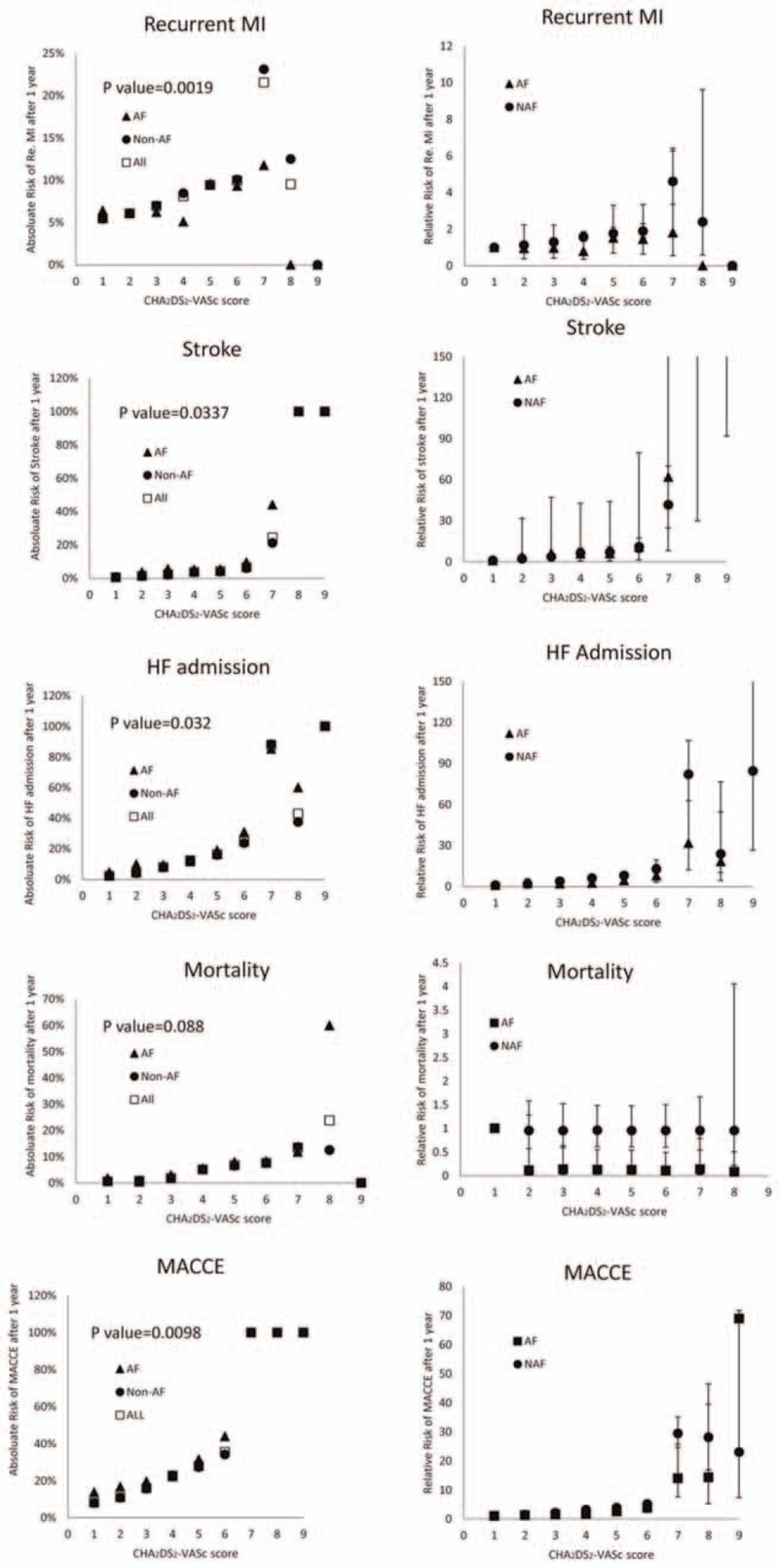
Incidences and relative risks stratified by CHA_2_DS_2_-VASc score and MACCE during 1-year follow-up. The absolute risks of different cardiovascular outcomes at 1 year in surviving patients with CHA_2_DS_2_-VASc scores from 1 to 9 are shown on the left. Unadjusted relative risks of different cardiovascular outcomes at 1 year for a CHA_2_DS_2_-VASc score of 1 are shown on the right. AF = atrial fibrillation, HF = heart failure, MACCE = major adverse cardiocerebral vascular event.

Since CHADS_2_ and CHA_2_DS_2_-VASc scores reportedly predict cardiovascular outcomes in ACS patients,^[12]^ we calculated the incidence of cardiovascular events and the relative risk of cardiovascular outcomes according to CHA_2_DS_2_-VASc score. The objective was to determine whether CHA_2_DS_2_-VASc scores can be used as prognostic indicators for AMI survivors with or without AF. Figure 3 shows that, in both strata, the 1-year incidence of cardiovascular events generally increased as CHA_2_DS_2_-VASc scores increased. However, the low incidence of recurrent MI, HF admission, and mortality in patients with CHA_2_DS_2_-VASc scores of 8 to 9 may have been due to the low number of patients. Comparisons of relative risk between patients with scores of 1 and patients with scores >1 showed that CHA_2_DS_2_-VASc scores had strong associations with stroke, HF admission, and MACCE. The linear-log regression model showed significantly increased incidences of all cardiovascular events and MACCE but not mortality. Notably, our study showed that patients with non-fatal MI (with or without AF) who had CHA_2_DS_2_-VASc scores of 7 to 9 had an incidence of 100% in MACCE within 1 year after discharge.

Table 4 compares the mean CHA_2_DS_2_-VASc scores in patients with and without MACCE. The discriminatory power of the CHA_2_DS_2_-VASc score is also shown. Regardless of the type of cardiovascular event, patients with MACCE within 1 year after discharge had significantly higher CHA_2_DS_2_-VASc scores compared with those without MACCE (*P* < .001). The C statistic at 1-year follow-up also predicted various endpoints. Notably, the area under the curve for HF admission and mortality exceeded 0.70. When using sensitivity and specificity to select cut-off points for predicting MACCE, a CHA_2_DS_2_-VASc score of <5 had negative predictive values of 93.37% for recurrent MI, 98.45% for stroke, 94.86% for HF admission, 98.83% for mortality, and 87.80% for MACCE. These data indicated that the CHA_2_DS_2_-VASc score is useful for discriminating between non-fatal MI patients who are and are not expected to experience MACCE after discharge.

**Table 4:**
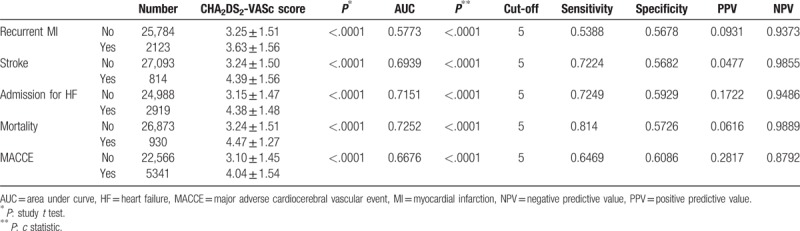
Discriminatory power of CHA_2_DS_2_-VASc score for cardiovascular outcomes at 1 year.

**Figure 4:**
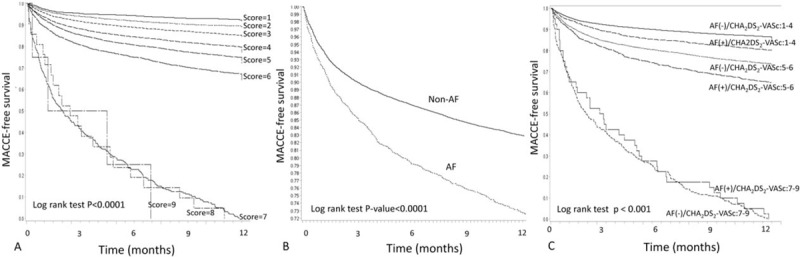
Kaplan survival estimates of 1-year MACCE-free survival stratified by AF rhythm and CHA_2_DS_2_-VASc score. Kaplan survival estimates of MACCE-free 1-year survival are stratified by presence or absence of new-onset AF and by CHA_2_DS_2_-VASc scores of 1 to 4, 5 to 6, and 7 to 9 (6 groups). MACCE = major adverse cardiocerebral vascular event.

MACCE-free survival estimates of patients with different CHA2DS2-VASc scores is demonstrated in Fig. 4A, showing gradually decreased MACCE-free survival rate along with the increase of CHA2DS2-VASc from 1 to 6, and MACCE-free survival in those with CHA2DS2-VASc ≥7 was 0% at 1 year (log rank test *P* < .001). Additionally, patients with AF had a lower MACCE-free survival at 1 year as compared with those without AF (log rank test *P* < .01) (Fig. 4B). Based on the result of our previous discrimination test, patients with CHA_2_DS_2_-VASc scores of 1 to 4, 5 to 6, and 7 to 9 were then divided into 3 subgroups with and without AF, which resulted in 6 subgroups. Figure 4C shows the results for the Kaplan–Meier survival analysis of 1-year MACCE in the 6 groups. The results showed that, for a given CHA_2_DS_2_-VAsc score group, outcomes were worse in non-fatal MI patients with AF than in those without AF. Importantly, the group with high CHA_2_DS_2_-VASc scores had a significantly higher incidence of MACCE at 1 year (log rank test *P* < .001). Furthermore, patients with CHA_2_D_2_-VASc scores of 5 to 6 had a much higher 1-year cumulative incidence of MACCE compared with those with CHA_2_D_2_-VASc scores of 1 to 4. Finally, almost all patients with CHA_2_D_2_-VASc scores of ≥7 had been admitted either for MI, HF, or stroke within 1 year after the index event regardless of the presence of AF.

## 4 Discussion

Our study had some interesting findings. First, this study of national health insurance claims data for patients with non-fatal MI showed that AF may negatively affect in-hospital and long-term prognosis. The CHA_2_DS_2_-VASc score has a strong correlation with MACCE, especially stroke and admission for HF. Secondly, patients with non-fatal MI and CHA_2_DS_2_-VASc scores of 7 to 9 were highly likely with MACCE within 1 year after discharge regardless the presence of AF. To our knowledge, this study is the first large population-base study to evaluate the use of CHA_2_DS_2_-VASc score for predicting MACCE in a population of patients with non-fatal MI.

### 4.1 AF is associated with poor MACCE outcomes in AMI patients

The incidence of AF, which is a common complication of AMI, is reportedly as high as 20%.^[13]^ Compared with AMI patients without AF, those with AF have a higher risk of in-hospital and long-term MACCE.^[14,15]^ Notably, a previous nationwide study showed that AF is associated with increased mortality in patients with first-time MI.^[16]^ The CHADS_2_ score is also a predictor of new-onset AF in AMI patients.^[12,17]^ A principal finding of our study was that the relative risks of MACCE within 1 year were significantly higher in the AF group than in the non-AF group. Additionally, compared with patients in the non-AF group, patients in the AF group tended to be older and have a higher incidence of cardiovascular disease before the AMI event, which suggests that AF is a hallmark of poor prognosis in AMI patients. Interestingly, the non-AF group had significantly more men and significantly higher incidences of dyslipidemia and statin use compared with AF group. Previous study showed that statin use was associated with lower incidence of new-onset AMI. Further studies will be needed to confirm our results.

### 4.2 CHA_2_DS_2_-VASc score for predicting AMI outcome

The CHA_2_DS_2_-VASc score is a risk index for predicting stroke in AF patients and can be used to guide anticoagulation therapy in AMI patients with AF.^[18]^ Additionally, the CHADS_2_ score is reportedly a strong independent predictor of future MACCE in AMI patients.^[19]^ The major strengths of this study are the use of hard data for cardiovascular outcomes and the large nationwide sample of AMI patients. One important finding is that, regardless of whether the patient has AF, the absolute incidences of recurrent MI, stroke, HF admission, and mortality have a linear correlation with CHA_2_DS_2_-VASc scores of 1 to 6. Additionally, non-fatal MI patients with CHA_2_DS_2_-VASc scores of ≥7 are likely to suffer MACCE within 1 year after discharge. These findings are consistent with a study of Taiwan Acute Coronary Syndrome Full Spectrum Registry data for 3183 patients with unstable angina or AMI, in which the endpoints were stroke, MI, and death.^[10]^ Since several components of the CHA_2_DS_2_-VASc score (age, women sex, DM, and prior stroke, or vascular disease) are prognostic predictors for AMI and stroke,^[20,21]^ the CHA_2_DS_2_-VASc score was expected to have clinical utility for identifying high-risk patients. A recent study also showed that CHA_2_DS_2_-VASc score is associated with risk of ischemic stroke, thromboembolism, and death in HF patients with or without AF.^[22]^ Together, the data obtained in this study indicate that the CHA_2_DS_2_-VASc score can be used to predict clinical outcomes in patients with AMI and HF. However, further studies are needed to compare the accuracy of CHA_2_DS_2_-VASc score with scoring systems with include biochemistry components such as TIMI risk and GRACE score.

### 4.3 Antithrombotic treatment in AMI patients with AF

The Swedish Heart Intensive Care Admissions Study revealed that oral anticoagulants (OACs) were prescribed in only 30% of AMI patients, even though OAC was associated with a 29% relative reduction and 7% absolute reduction in 1-year mortality.^[23]^ Current guidelines recommend dual anti-platelet therapy (DAPT) combined with OAC (triple therapy) as the initial antithrombotic therapy for AMI patients with AF.^[18]^ However, most elderly AMI patients with AF but without ST elevation who undergo percutaneous coronary intervention with stent placement receive DAPT rather than triple therapy at discharge.^[24]^ Similarly, although patients with AF had an average CHA_2_DS_2_-VASc score of 3.81 ± 1.47 in our patient cohort, warfarin was prescribed in only 18.1%, which suggests an under-treatment of these patients during 2008 to 2011 when novel oral anti-coagulants were unavailable in Taiwan. The PIONEER AF-PCI and REDUAL-PCI trials will reveal the best antithrombotic therapy for patients who undergo PCI as revascularization treatment for ACS.

### 4.4 Clinical impact of this study

This study showed that CHA_2_DS_2_-VASc score had a moderately high 1-year NPV for identifying non-fatal MI patients at “low risk” for recurrent MI, stroke, HF admission, or death (approximately 95%). In contrast, all patients with CHA_2_DS_2_-VASc scores of ≥7 had MACCE within 1 year regardless of AF. Therefore, patients with these scores should be considered very high risk patients and should be managed aggressively according to established guidelines. Recently, some studies also found CHA2DS2-VASc score predicted for failed reperfusion after thrombolytic therapy in patients with ST-elevation myocardial infarction, occurrence of AMI and cardiovascular outcome in patients with atrial fibrillation. Traditionally, the GRACE and TIMI score method are useful tools for predicting the clinical outcome in patients with AMI. GRACE and TIMI score system are more complicated and both scoring system all needed physiological and laboratory data to calculate. Comparing to GRACE and TIMI score, the CHA_2_DS_2_-VASc scoring system is a simple and easy tool to calculate without any information of physiological findings, laboratory data, and information of drug using. Therefore, CHA_2_DS_2_-VASc may be a comprehensive, convenience, and simple method for clinical physician in risk evaluation of patients with AMI.

### 4.5 Study limitations

The major limitation of this study is that ICD-9-CM codes were used to define cardiovascular endpoints. Therefore, coding errors in this claims database are expected even though the analysis was limited to patients discharged alive after the index event. Additionally, since the population analyzed in this study excluded patients with fatal MI, the accuracy of outcome predictions based on CHA_2_DS_2_-VASc scores was not evaluated in this patient population. Finally, unmeasured confounders such as medications may exist, and only associations between variables were studied.

## 5 Conclusion

In patients with non-fatal AMI, 1-year cardiovascular outcomes are worse in those with AF compared with those without AF. Regardless of the presence of AF, the CHA_2_DS_2_-VASc score is an accurate predictor of 1-year MACCE in AMI patients.

## Author contributions

**Conceptualization:** Wen-Jung Chung.

**Data curation:** Chen-Yu Li, Cheng-Jui Lin Lin, Cheng-I Cheng.

**Formal analysis:** Chen-Yu Li, Chee-Jen Chang, Cheng-Jui Lin Lin, Tzu-Hsien Tsai.

**Investigation:** Wen-Jung Chung, Chiung-Jen Wu, Cheng-I Cheng.

**Methodology:** Chen-Yu Li, Chee-Jen Chang, Wen-Jung Chung.

**Project administration:** Chee-Jen Chang.

**Software:** Shu-Kai Hsueh, Chien-Ho Lee.

**Supervision:** Cheng-I Cheng.

**Validation:** Shu-Kai Hsueh, Chien-Ho Lee.
